# Supplementary material for: Mapping the evolution of fertility support policies in China: A content and instrumental analysis
Source: PLoS One. 2025 Oct 9;20(10):e0332137. doi: 10.1371/journal.pone.0332137 (PMC12510515; doi:10.1371/journal.pone.0332137)
Supplement: S1 Appendix — (ZIP) [file pone.0332137.s001.zip › S1 Appendix. 226 original policy documents/193-国家发展改革委、民政部、国家卫生健康委关于印发《“十四五”积极应对人口老龄化工程和托育建设实施方案》的通知(FBM-CLI-4-5015949).doc]

国家发展改革委、民政部、国家卫生健康委关于印发《“十四五”积极应对人口老龄化工程和托育建设实施方案》的通知

**发布部门：** [国家发展和改革委员会(含原国家发展计划委员会、原国家计划委员会)](https://www.pkulaw.com/law/chl?Aggs.IssueDepartment=60301) [民政部](https://www.pkulaw.com/law/chl?Aggs.IssueDepartment=60208) [国家卫生健康委员会](https://www.pkulaw.com/law/chl?Aggs.IssueDepartment=60321) [机构沿革](javascript:void(0))

**发文字号：**发改社会〔2021〕895号

**发布日期：**2021.06.17

**实施日期：**2021.06.17

**时效性：** [现行有效](https://www.pkulaw.com/law/chl?Aggs.TimelinessDic=01)

**效力级别：** [部门规范性文件](https://www.pkulaw.com/law/chl?Aggs.EffectivenessDic=XE0302)

**法规类别：** [老少妇幼残保护](https://www.pkulaw.com/law/chl?Aggs.Category=01604)

国家发展改革委、民政部、国家卫生健康委关于印发《“十四五”积极应对人口老龄化工程和托育建设实施方案》的通知
（发改社会〔2021〕895号）

各省、自治区、直辖市及计划单列市、新疆生产建设兵团发展改革委、民政厅（局）、卫生健康委，北大荒农垦集团有限公司：
　　为推进实施积极应对人口老龄化国家战略，以“一老一小”为重点完善人口服务体系，扩大养老托育服务有效供给，提升服务质量，完善服务体系，不断满足人民日益增长的美好生活需要，根据《[中华人民共和国国民经济和社会发展第十四个五年规划和2035年远景目标纲要](https://www.pkulaw.com/chl/dad03bd96074290cbdfb.html)》和中共中央、国务院印发的《国家积极应对人口老龄化中长期规划》、《[国务院办公厅关于促进养老托育服务健康发展的意见](https://www.pkulaw.com/chl/d1ad8fb179db9915bdfb.html)》（国办发〔2020〕52号）等有关文件要求，国家发展改革委、民政部、国家卫生健康委共同制定了《“十四五”积极应对人口老龄化工程和托育建设实施方案》（以下简称《实施方案》）。
　　现印发你们，请结合《积极应对人口老龄化工程和托育建设中央预算内投资专项管理办法》（发改社会规〔2021〕525号）要求，认真做好项目前期准备工作，加强对《实施方案》实施的监督检查，确保建设质量，提高资金使用效益，适时开展评估工作。

国家发展改革委
　　民　政　部
　　国家卫生健康委
　　2021年6月17日

　　[附件：《“十四五”积极应对人口老龄化工程和托育建设实施方案》](https://resources.pkulaw.cn/staticfiles/fagui/20210625/17/19/0/3ee62de6298ed3a0ea4edd47cbf05000.pdf)

© 北大法宝：（[www.pkulaw.com](https://www.pkulaw.com/)) 专业提供法律信息、法学知识和法律软件领域各类解决方案。北大法宝为您提供丰富的参考资料，正式引用法规条文时请与标准文本核对。
欢迎查看所有[产品和服务](https://www.pkulaw.com/helps/2.html)。法宝快讯：[如何快速找到您需要的检索结果？](https://www.pkulaw.com/helps/70.html)[法宝](https://www.pkulaw.com/helps/69.html)V6有何新特色？

扫描二维码阅读原文

**原文链接：**<https://www.pkulaw.com/chl/55b5d19d54f62733bdfb.html>
